# Comment on: A Systematic Review and Meta-Analysis of the Association Between ACTN3 R577X Genotypes and Performance in Endurance Versus Power Athletes and Non-Athletes

**DOI:** 10.1186/s40798-025-00874-1

**Published:** 2025-06-02

**Authors:** Ricardo Muller Bottura

**Affiliations:** 1 Department of Neuroscience, Rehabilitation, Ophthalmology, Genetics, and Maternal-Infant Sciences (DINOGMI), Università degli Studi di Genova, Genoa, Italy; 2 Department of Neurosciences, Biomedicine and Movement Sciences, Università degli Studi di Verona, Verona, Italia

Dear Editor, 

I read with great satisfaction the systematic review on the association between the *ACTN3 R577X* polymorphism and performance [1]. I would like to comment that, although I appreciated every paragraph, as this topic is crucial for those working in training today, I feel obligated to point out something that I consider to be a significant error in the paper’s conclusion. Since the publication of Yang et al. [2], the same considerations have been presented erroneously, namely that athletes with the RR genotype would have an advantage in strength/power sports. In my view, and that of those who work with me daily, applying genetic information to training (whether in strength/power or endurance sports), the correct conclusion is somewhat different: XX athletes do *not* have an advantage in strength/power sports. It may seem like a minor distinction, but this reversal of facts is crucial, and I will explain why. I start with the analysis presented in Table 3 of the article [1], regarding the percentage distribution of *ACTN3 R577X* genotypes [1]. It shows that, both in strength/power and endurance sports, there is a greater representation of athletes with the RR genotype compared to the control group, even though the representation of XX athletes remains equal in the endurance group compared to the control group [1]. However, this does not indicate an advantage for XX athletes in endurance, as there is no evidence that they can sustain extreme endurance performance [3]. Furthermore, several studies show a greater representation of athletes with the RR genotype in endurance sports [4, 5]. The most compelling evidence highlighting the significance of ACTN3, the R allele, and the RR genotype in endurance sports is their prevalence among Kenyans and Ethiopians [6]. We have recently witnessed the breaking of Olympic records in the Paris 2024 Marathon by two Ethiopian athletes (although the female athlete is a naturalized Dutch citizen), whose population has about 92% with the RR or RX genotype. Last year, we also saw world marathon records broken by Tigist Assefa (Ethiopian) in the women’s category and the late Kelvin Kiptum (Kenyan) in the men’s category. These records were previously held by two Kenyans, whose R allele prevalence reaches 99%, with 84% being RR. The quest for a sub-2-hour marathon (achieved in a simulation by Kenyan Eliud Kipchoge), along with noticeable performance increases in other endurance sports (as demonstrated by Tadej Pogacar in the Tour de France), further emphasizes the need for a muscle structure that supports not just the final result, but the daily training, allowing for better recovery, adaptation, and a lower risk of injury. This is precisely what ACTN3 provides [7]. In conclusion, while I commend the excellent work of this meta-analysis, I suggest that future studies emphasize, especially for those not yet familiar with the practical application of genetics in sports, that expressing ACTN3 is advantageous for both extremes of sports, whether strength/power or endurance.

## Data Availability

Not applicable.

## Abbreviations

ACTN3: Alpha-actinin-3
ACTN3 R577X: ACTN3 gene polymorphism
R allele: Allele of the ACTN3 gene polymorphism associated with ACTN3 production
RR: Homozygous ACTN3 genotype associated with ACTN3 production
RX: Heterozygous ACTN3 genotype
XX: Homozygous ACTN3 genotype that does not produce ACTN3
